# The Principles and Practice of Dentistry

**Published:** 1889-07

**Authors:** 


THE PRINCIPLES AND PRACTICE OF DENTISTRY.—When we received this book, we placed it beside former editions—those of Harris, Austen and Gorgas—for the purpose of comparison as to size. Until the eleventh edition, the book contained but a few over seven hunured pages, in fact that number of pages was ample to record all there was of Dental science and practice, but this edition contains over *twelve hundred pages*. Nothing further is needed to convince the incredulous of the enormous advance in the science and art of dentistry than the necessity of publishing such a mammoth volume. This increase in size has been made by addition to almost every chapter, and by the insertion of new matter, not as a pad to swell the volume to unwieldy proportions, but because it was absoluely necessary in order to include the recent discoveries and latest approved systems. It is indeed "A complete encyclopædia of the science and art of dentistry." We consider this editon of Harris' the most valuable book for dentists and students that has been published in several years. Cherish the old editions for the benefit they have been to your professional knowledge, but procure this new edition for instruction in modern practice. Every class of work in the province of the progressive dentist is described in this truly great work. It is gratifying to know that "Harris' Principles and Practice" is used as a text-book in every civilized country, and has been translated into several languages.

It may be of interest to know that the 1st edition of Harris was published in 1839, under the title of "The Dental Art: a Practical Treatise on Dental Surgery," and contained three plates. The 2d edition was published in 1844, the title being "The Principles and Practice of Dental Surgery," The 3d edition in 1848; the 4th in 1850; the 5th in 1852, the 6th in 1855; the 7th in 1858; the 8th in 1863; the 9th in 1866; the 10th in 1871, edited by Dr. Philip H. Austen; the 11th in 1885, edited by Dr. F. J. S. Gorgas, when the title was changed to "The Principles and Practice of Dentistry," which title is retained in the present or 12th edition.—*Dental Advertiser.*

